# *RB1CC1* duplication and aberrant overexpression in a patient with schizophrenia: further phenotype delineation and proposal of a pathogenetic mechanism

**DOI:** 10.1002/mgg3.1561

**Published:** 2020-12-19

**Authors:** Edoardo Errichiello, Roberto Giorda, Antonella Gambale, Achille Iolascon, Orsetta Zuffardi, Sabrina Giglio

**Affiliations:** ^1^ Unit of Medical Genetics Department of Molecular Medicine University of Pavia Pavia Italy; ^2^ Laboratory of Molecular Biology Scientific Institute IRCCS Eugenio Medea Bosisio Parini Italy; ^3^ Department of Molecular Medicine and Medical Biotechnologies University "Federico II" of Naples Naples Italy; ^4^ CEINGE, Advanced Biotechnologies Naples Italy; ^5^ Unit of Medical Genetics, Department of Medical Sciences and Public Health University of Cagliari Cagliari Italy

**Keywords:** autophagy, copy number variations (CNVs), *RB1CC1*, schizophrenia (SCZ), suicidality

## Abstract

**Background:**

Copy number variants in coding and noncoding genomic regions have been implicated as risk factor for schizophrenia (SCZ). Rare duplications of the *RB1CC1* gene were found enriched in SCZ patients. Considering that the effect of such duplications on *RB1CC1* expression has never been evaluated and partial gene duplications of *RB1CC1* have also been reported in SCZ patients, it is unclear whether the pathogenesis is mediated by haploinsufficiency rather than genuine overexpression of the gene.

**Methods and Results:**

We studied a patient with schizophrenia, suicidality, and obesity, who carried a de novo *RB1CC1* complete duplication, as assessed by high‐resolution array‐CGH. Molecular breakpoint cloning allowed to identify nonhomologous end joining (NHEJ) as driving mechanism in this rearrangement. On the contrary, trio‐based whole‐exome sequencing excluded other potential causative variants related to the phenotype. Functional assays showed significant overexpression of *RB1CC1* in the peripheral blood lymphocytes of the proband compared to control subjects, suggesting overdosage as leading mechanism in SCZ pathophysiology.

**Conclusion:**

We hypothesized a pathogenetic model that might explain the correlation between *RB1CC1* overexpression and schizophrenia by altering different cell signaling pathways, including autophagy, a promising therapeutic target for schizophrenic patients.

## Graphical abstract

It is unproven whether the RB1CC1 pathogenesis in schizophrenia is mediated by haploinsufficiency rather than genuine overexpression of the gene. We identified a de novo RB1CC1 complete duplication in a SCZ patient showing significant overexpression of RB1CC1. We hypothesized a pathogenetic model that might explain the correlation between RB1CC1 overexpression and schizophrenia by altering different cell signaling pathways, including autophagy, a promising therapeutic target for schizophrenic patients.

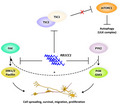

## 1 INTRODUCTION

Copy number variants (CNVs), frequently occurring de novo, have been implicated in the genetic etiology of schizophrenia (SCZ) (Bassett et al., 2017; Buizer‐Voskamp et al., 2011; Clifton et al., 2017; D'Angelo et al., 2016; Glessner et al., 2017; Hippolyte et al., 2016; Maillard et al., 2015; Marshall et al., 2017; Stefansson et al., 2009). Noncoding CNVs could also contribute to the genetic vulnerability to the disorder by affecting regulatory promoters and enhancer elements (Fullard et al., 2017; Tansey & Hill, 2018; Won et al., 2016).

Duplications at chromosome 8q11.23, including *RB1CC1* (RB1‐inducible coiled‐coil 1; OMIM *606837), all with different breakpoint boundaries, have been reported in 9/8461 patients and 14/11,2871 control individuals screened by a genome wide single‐nucleotide polymorphism (SNP) array, highlighting a significant association with SCZ, accompanied in some cases by suicidality (Degenhardt et al., 2013). Complete and partial *RB1CC1* gains have also been reported in a few patients with intellectual disability (ID) and/or developmental delay (Cooper et al., 2011), and autism spectrum disorder (ASD) (Marshall et al., 2008).

In this study, we characterized a duplication at the 8q11.23 region involving *RB1CC1* by using a combination of high‐resolution array‐CGH and breakpoint cloning. Furthermore, we provided functional evidence of *RB1CC1* overexpression, which likely mediates SCZ pathogenesis through different paths, including autophagy, which is considered as a guardian against neurodegeneration and a druggable target in schizophrenic patients.

## 2 MATERIALS AND METHODS

### 2.1 Editorial policies and ethical considerations

This study was conducted in accordance with the Declaration of Helsinki and national guidelines. Written informed consent for participation and publication was obtained from all subjects.

### 2.2 Clinical description

The patient, a 20‐year‐old male, was born after a pregnancy complicated by gestosis during the second–third trimesters. He showed clumsy, uncoordinated gait, and speech delay since the age of 2.5 years. Clinical evaluation at 4 years ascertained mild psychomotor delay, memory impairment, bulimia, obesity (BMI >45), hepatomegaly, nuchal small fibromas, and fecal incontinence. Brain MRI was normal. He started developing psychotic episodes and self‐injury (hanging/asphyxiation) at 12 years. Neuropsychological assessment revealed aggressive/suicidal behavior, obsessive‐compulsive disorder, extremely low frustration tolerance, sleep disturbance (despite benzodiazepine administration), and hypoalgesia (ICD‐10‐CM: F06.0).

Because of neurological features and obesity, he was first diagnosed with Smith–Magenis syndrome (OMIM #182290), which was excluded after *RAI1* (*607642) negative testing.

### 2.3 Array‐CGH

Molecular karyotyping was performed by using a high‐density 400 K chip (Agilent), according to manufacturer's protocol. Data were analyzed by using the Agilent Genomic Workbench Standard Edition 6.5.0.58, as previously described (Errichiello et al., 2016). Genomic coordinates are reported according to the GRCh38/hg38 genome assembly.

### 2.4 Trio whole‐exome sequencing (trio‐WES)

Whole‐exome sequencing was performed on the DNA isolated from a peripheral blood sample of the patient and his parents by using the QIAamp DNA Blood Mini Kit (Qiagen), according to the manufacturer's instructions. Libraries were generated using a commercial target enrichment kit (SureSelect Human All Exome V7, Agilent Technologies), and sequenced on a HiSeq 2500 sequencing platform (paired‐end 2 × 100 bp; Illumina), as previously reported (Errichiello et al., 2017). Annotation was carried out with ANNOVAR and only variants with a minimum quality score of 20 and a minimum read depth of 10× were included in the downstream analysis.

In the bioinformatic analysis were excluded variants reported in gnomAD v2.1.1, TOPMed, ExAC, 1000 Genomes, and NHLBI ESP6500, and in‐house database (composed of approximately 1500 individuals), with a frequency above 5% and outside exonic or splice site (beyond 30 bp of exon/intron boundaries) regions. After a preliminary variant filtering focused on a virtual panel of clinically relevant genes implicated in SCZ (Table S1), NGS data were further filtered according to possible inheritance patterns. CNV analysis was performed by using the Control‐FREEC and EXCAVATOR tools.

### 2.5 Cloning of the duplication breakpoints

Q‐PCR reactions (PowerUp MasterMix PCR System, Applied Biosystems) were performed on genomic DNA to refine the breakpoints’ location by using specific probes for the distal and proximal breakpoint regions (available upon request). Then, long‐range PCR (JumpStart AccuTaq LA PCR, Sigma‐Aldrich) was set up to sequence the junction fragment on a 3500/3500xl Genetic Analyzer (Applied Biosystems).

### 2.6 RB1CC1 expression analysis

*RB1CC1* expression on the peripheral blood lymphocytes (PBLs) was measured by qRT‐PCR of random primer‐synthetized proband's cDNA (iScript cDNA Synthesis Kit, Bio‐Rad) against eight control PBL cDNAs using a specific TaqMan assay (Hs01089002_m1, Applied Biosystems). A *GAPDH* probe (Hs99999905_m1, Applied Biosystems) was used as housekeeping gene control. All assays were performed on a QuantStudio 3 instrument (Applied Biosystems).

## 3 RESULTS

**FIGURE 1:**
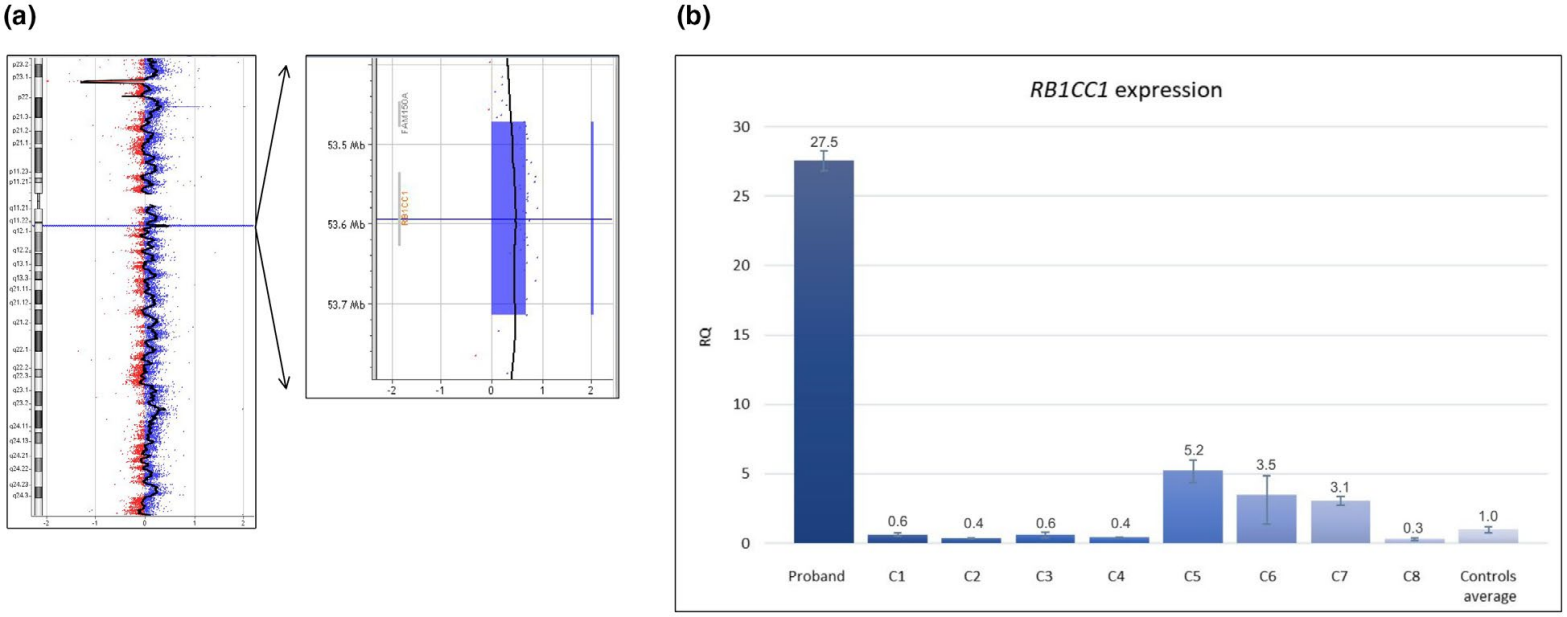
*RB1CC1* duplication and overexpression. (a) Identification of *RB1CC1* duplication by high‐resolution array‐CGH (400 K). The duplicated region, arr[GRCh38] 8q11.23(52560156_52801994)x3, encompassing *RB1CC1*, does not completely overlap with any CNV reported in DGV (Database of Genomic Variants), and does not disrupt any topologically associating domain (TAD), as assessed by 3D Genome Browser (http://promoter.bx.psu.edu/hi‐c/). The duplication was also confirmed on NGS data by using the Control‐FREEC and EXCAVATOR CNV‐calling tools. (b) *RB1CC1* expression in patient and controls. *RB1CC1* expression in proband's PBLs, adjusted for variable cDNA amount measured by *GAPDH* expression, was over 27 times higher than the average of eight healthy controls without *RB1CC1* CNVs. All samples were run in triplicate

High‐resolution array‐CGH detected a de novo heterozygous germline duplication at the 8q11.23 locus, as also assessed by Control‐FREEC/EXCAVATOR and IGV visual inspection on the NGS data (Figure 1a and Figure S1a), which arose on the maternal allele (Figure S1b). In contrast, neither CNVs nor variants were detected in the *RAI1* gene, which was suspected to be the culprit gene on clinical grounds. Breakpoint analysis refined the duplicated region to 252,244 bp (chr8:52,555,810–52,808,053), spanning the entire *RB1CC1* gene and the first exon of *ALKAL1*/*FAM150A* (Figure 1a and Table S2). Similar duplications are reported in the Database of Genomic Variants (DGV), as well as in DECIPHER patients with mainly neurodevelopmental disorders. Sequencing of the proband‐specific LR‐PCR fragment revealed a junction between two unrelated LINE‐1 repeated DNA sequences and a 1‐bp microhomology, consistent with a nonhomologous end joining (NHEJ) mechanism (Table S2). As a consequence of this duplication, *RB1CC1* expression in proband's PBLs was over 27 times higher than the average of control samples (Figure 1b), possibly due to the perturbation of the negative feedback loop mechanism of the *RB1CC1* transcription (Loehlin & Carroll, 2016).

On the contrary, trio‐WES failed to identify potential candidate variants in genes associated with patient's neurophenotype, further strengthening the causative role of *RB1CC1* duplication. Notably, the only variant related to SCZ was a maternally inherited hemizygous missense substitution in *HS6ST2* (*300545) on chromosome Xq26.2: NM_001077188.2:c.347C>T, NP_001070656.1:p.(Thr116Ile) (rs370454722). However, three European Non‐Finnish hemizygotes are listed in gnomAD v2.1.1, whereas Piton et al. (2011) identified a *HS6ST2* truncating variant in a healthy XY individual, suggesting “male tolerance” and possible functional redundancy with other heparan sulfate 6‐O‐sulfotransferase isoforms. Based on this evidence, we excluded a pathogenetic role of this variant, which was also classified as likely benign according to the ACMG guidelines. The molecular and clinical details of our patient have been submitted in the ClinVar database (#VCV000544682.1).

## 4 DISCUSSION

*RB1CC1* duplications have been detected at low frequency in large cohorts of SCZ patients as well as in control subjects, as expected for a disorder characterized by remarkable genetic heterogeneity and reduced penetrance, due to the likely combination of CNVs and susceptibility alleles (Richards et al., 2016). It is reasonable that the contribution of rare germline variants in the complex SCZ genomic architecture, including structural variants affecting the boundaries of topologically associated domains (TADs), will spread thanks to more extended whole‐genome sequencing studies on large cohorts of patients (Halvorsen et al., 2020).

In DECIPHER are currently listed 46 individuals with a CNV gain but only one patient with a CNV loss spanning the *RB1CC1* locus. The duplication involves the *RB1CC1* gene without affecting any other known disease‐causing gene in 28 patients, of whom 18 with complete duplication and 10 with partial duplication. Most of these individuals developed ID, whereas ASD and delayed speech and language development are reported in five and two of them, respectively. In case #257475, a 20‐year‐old male, hyperactivity, short attention span, and truncal obesity have been also observed. However, it may be speculated that SCZ or SCZ‐like features might be underrepresented in DECIPHER, as the median age of RB1CC1‐duplicated cases is around 6 years, when the SCZ clinical diagnosis is challenging. The effect of such duplications on *RB1CC1* gene expression has never been evaluated in CNV carriers and, since partial gene duplications of *RB1CC1* have also been documented in schizophrenic subjects, it is unproved whether the pathomechanism is mediated by haploinsufficiency due to gene disruption rather than genuine overexpression of the gene. Notably, Degenhardt et al. (2013) reported full *RB1CC1* duplication in three SCZ patients, partial gene duplication in five patients, and a duplication immediately upstream of the *RB1CC1* gene in an additional patient. Importantly, all partial gene duplications were detected by chromosomal microarray only without breakpoint‐level analysis, which is essential to interpret their effects on gene structure in terms of orientation, location, and possible alteration of the reading frame causing loss‐of‐function. In this regard, it has been shown that most genome duplications (83%) are tandem in direct orientation (head‐to‐tail adjacent to the original locus) and do not disrupt genes (Newman et al., 2015). Xu et al. (2011) identified a rare de novo frameshift variant [NM_014781.5:c.3682_3683delGA, NP_055596.3:p.(Glu1228ThrfsTer7); HGMD #CD119371] in a sporadic SCZ patient, theoretically supporting a loss‐of‐function mechanism. Although this variant is unreported in publicly available databases and multiple lines of computational evidence support its deleterious effect, it has not been functionally validated and, most importantly, behavioral disturbances have never been observed in conditional knockout mice (Gan et al., 2006; Wei et al., 2009; Yao et al., 2015). In this study, we documented the aberrant overexpression of *RB1CC1* in a schizophrenic patient with complete gene duplication. However, it cannot be ruled out that *RB1CC1* might be sensitive to both haploinsufficiency and triplosensitivity culminating in neurodevelopmental anomalies. Therefore, more functional investigations are needed to address this point.

A part from *RB1CC1*, our duplication encompassed *ALKAL1*/*FAM150A*, which encodes the ALK and LTK ligand 1, the physiological ligand (together with ALK and LTK ligand 2, a.k.a. ALKAL2) of Alk (Anaplastic lymphoma kinase) and Ltk (Leukocyte tyrosine kinase) receptor tyrosine kinases (RTKs) with demonstrated oncogenic potential (Reshetnyak et al., 2015). Mo et al. (2017) proved that Alk and Ltk ligands are essential for iridophore formation in the adult zebrafish eye. Therefore, although we did not measure the expression of *ALKAL1*/*FAM150A*, it is unlikely involved in the complex neurobehavioral phenotype observed in our as well as in other previously reported patients with CNV gains involving *ALKAL1*/*FAM150A*.

**FIGURE 2:**
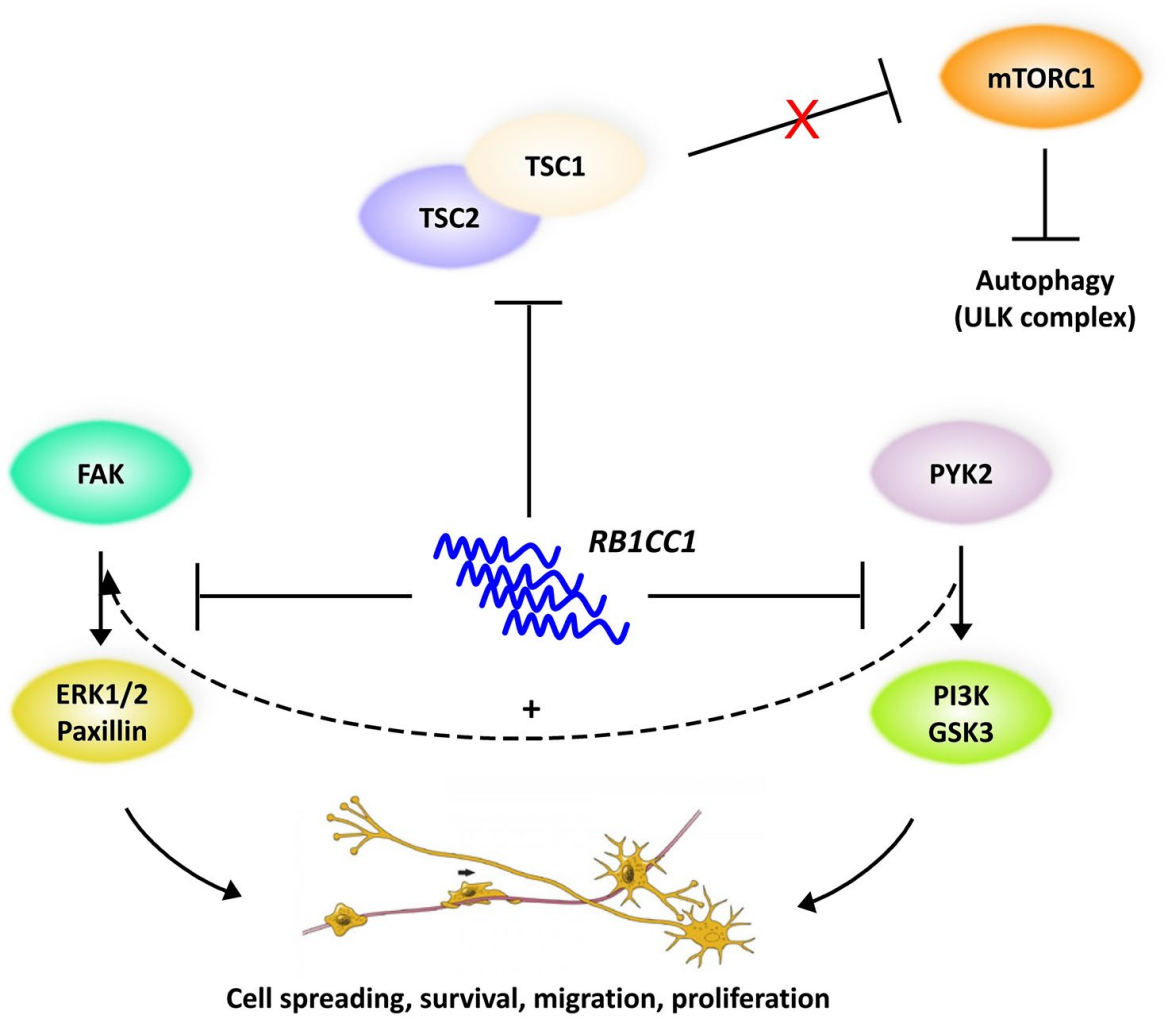
Cascade of events triggered by the overexpression of *RB1CC1* in SCZ pathogenesis. Duplication‐induced overexpression of RB1CC1/FIP200 inhibits FAK, which physiologically regulates cell spreading and motility upon FAK‐Src signaling complex formation and paxillin/ERK1/2 phosphorylation and activation. Upregulated RB1CC1 also blocks PYK2 tyrosine kinase activity upon PI3K/Akt pathway, which promotes cell survival and proliferation, and GSK3 signaling, which instead controls neurogenesis, neuronal polarization, and axon growth during brain development. Importantly, PYK2 indirectly enhances paxillin activation through ERK1/2 MAP kinases. Finally, overexpressed RB1CC1 interferes with TSC1–TSC2 complex assembly/stabilization, a critical negative regulator of mTORC1. MTORC1 controls anabolic processes to promote cell growth and, importantly, strongly prevents autophagy initiation by regulating the activity of the ULK1 complex that is required for the formation of autophagosomes. Thus, the lack of mTORC1 inhibition by TSC1/TSC2 finally leads to autophagy blockade and neurocytotoxicity

The brain‐expressed RB1CC1/FIP200 regulates a variety of cellular processes, including cell cycle progression, differentiation, senescence, apoptosis, neural migration/spreading, and neurodegeneration (Wang et al., 2013). Molecular studies on *RB1CC1* shed new light on the putative role of mTOR signaling pathway and autophagy in the pathogenesis of SCZ (Menzies et al., 2015; Merenlender‐Wagner et al., 2015), as supported by the previous finding that RB1CC1, together with ULK1 and ULK2 serine/threonine kinases that play a key role in autophagy induction, is involved in the regulation of axon guidance during brain development (Wang et al., 2017). Furthermore, rare variants in *ULK1* were found to be enriched in SCZ cases compared to controls (Al Eissa et al., 2018). Intriguingly, overexpression of RB1CC1/FIP200 was shown to inhibit FAK (Fan et al., 2013) and Pyk2 kinase activity (Abbi et al., 2002) as well as TSC1–TSC2 complex formation (Gan et al., 2005), which in turn negatively regulates mTORC1 (Di Nardo et al., 2014), a critical regulator of autophagy (Kim et al., 2011) (Figure 2). We speculated that aberrant *RB1CC1* mRNA expression might lead to decreased protein solubility and aggregation‐induced neurotoxicity, following the DISC1 pathogenic model (Atkin et al., 2012). Although confirmatory expression studies in postmortem brains or induced pluripotent stem cells (iPSC) of schizophrenic patients are needed, we suggest that *RB1CC1* upregulation might be considered as a tentative plasmatic biomarker for suicidality (Niculescu et al., 2017) and, most importantly, a druggable target in SCZ patients, as previously demonstrated for BECN1/Beclin 1 (Menzies et al., 2017; Merenlender‐Wagner et al., 2014).

## CONFLICT OF INTEREST

The authors declare no conflict of interest.

## AUTHOR CONTRIBUTIONS

E.E.: Conceptualization, Investigation, Writing ‐ Original Draft, Writing ‐ Review & Editing; R.G.: Investigation, Writing ‐ Review & Editing; A.G.: Investigation, Writing ‐ Review & Editing; A.I.: Writing ‐ Review & Editing, Funding acquisition, Supervision; O.Z.: Writing ‐ Review & Editing, Funding acquisition, Supervision; S.G.: Writing ‐ Review & Editing, Funding acquisition, Supervision.

## Supporting information

Table S1‐S2‐Fig S1
