# Supplementary material for: RB1CC1 duplication and aberrant overexpression in a patient with schizophrenia: further phenotype delineation and proposal of a pathogenetic mechanism
Source: Mol Genet Genomic Med. 2020 Dec 19;9(1):e1561. doi: 10.1002/mgg3.1561 (PMC7963413; doi:10.1002/mgg3.1561)
Supplement: Supplementary file 1 — Table S1‐S2‐Fig S1 [file MGG3-9-e1561-s001.docx]

**Table S1. Panel of clinically relevant genes associated with SCZ**

| Gene | Location | Gene OMIM |
| --- | --- | --- |
| *AKT1* | 14q32.33 | 164730 |
| *APOL2* | 22q12.3 | 607252 |
| *APOL4* | 22q12.3 | 607254 |
| *CHI3L1* | 1q32.1 | 601525 |
| *COMT* | 22q11.21 | 116790 |
| *DAO* | 12q24.11 | 124050 |
| *DAOA* | 13q33.2 | 607408 |
| *DISC1* | 1q42.2 | 605210 |
| *DISC2* | 1q42.2 | 606271 |
| *DRD3* | 3q13.31 | 126451 |
| *HTR2A* | 13q14.2 | 182135 |
| *MTHFR* | 1p36.22 | 607093 |
| *PRODH* | 22q11.21 | 606810 |
| *RBM12* | 20q11.22 | 607179 |
| *RTN4R* | 22q11.21 | 605566 |
| *SHANK3* | 22q13.33 | 606230 |
| *SLC1A1* | 9p24.2 | 133550 |
| *SYN2* | 3p25.2 | 600755 |

**Table S2. Characterization of duplication breakpoints**

| **Breakpoint** | **Chr. coordinates (hg38)** | **Sequence** | | | **Flanking repeat elements** |
| --- | --- | --- | --- | --- | --- |
| Proximal | chr8:52,555,750-52,555,810 | cttggctttcctgtggtaatcacagtacaa | c | ctcttgcccacattttccccgttcctcctt | L1MC3 (LINE, L1) |
| Junction |  | caccaccattatcaggatacagaattgttt | c | ctcttgcccacattttccccgttcctcctt |  |
| Distal | chr8:52,808,053-52,808,113 | caccaccattatcaggatacagaattgttt | c | ttcactaaaaagctccctcaggctgcctgt | L1ME1 (LINE, L1) |

**Figure S1. IGV visual inspection and unbalanced variant allele fraction (VAF) at the *RB1CC1* locus in the proband and parents.**

**
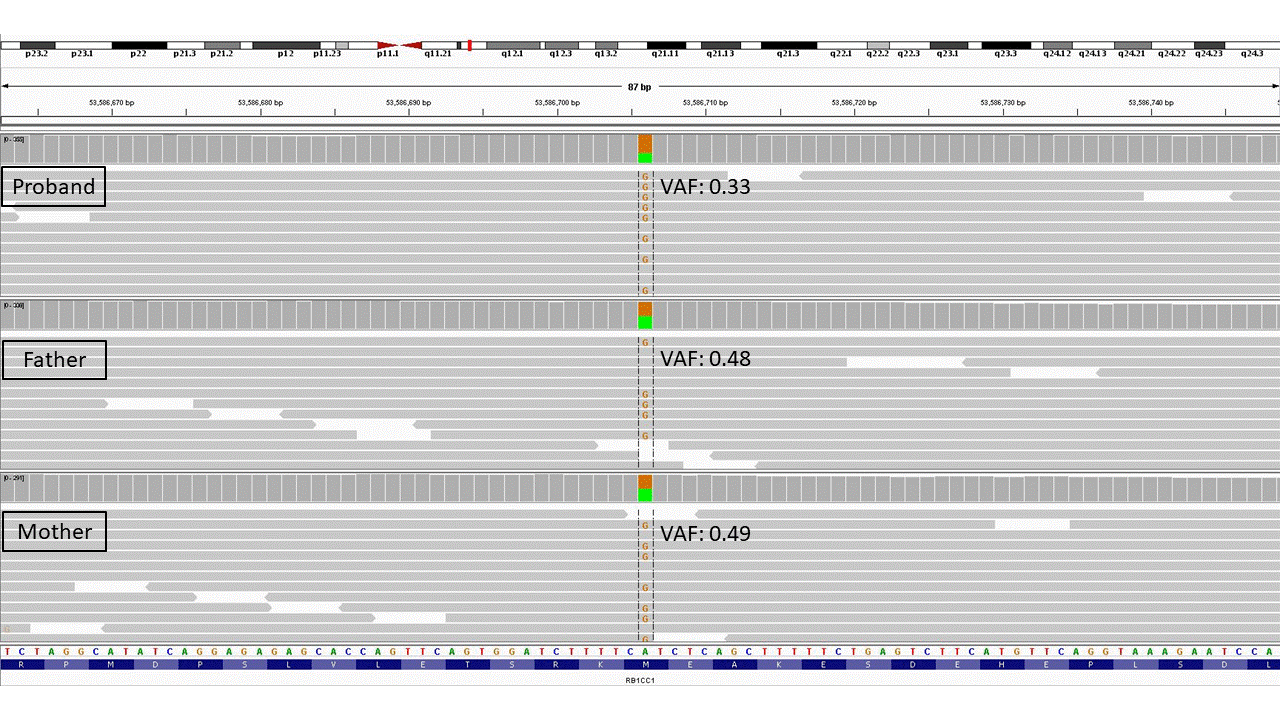
(a)**

**
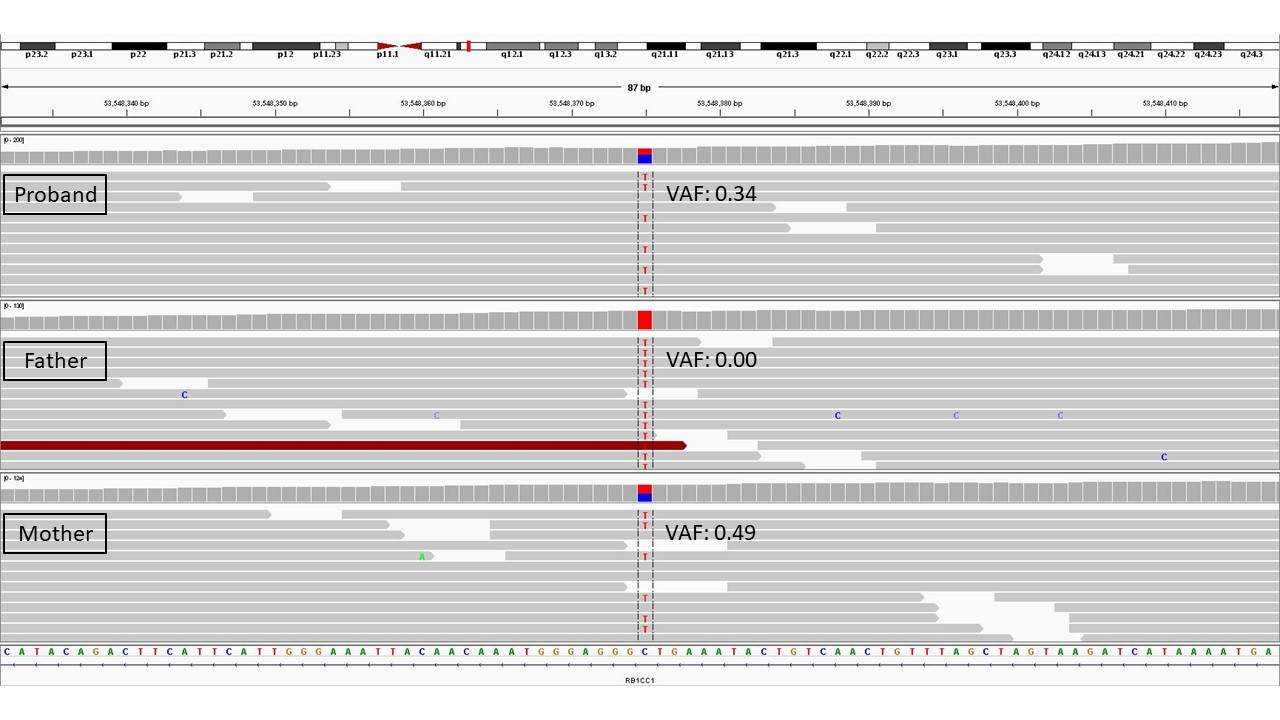
(b)**
